# The Pittsburgh Sleep Quality Index: a brief review

**DOI:** 10.1093/occmed/kqae121

**Published:** 2025-04-05

**Authors:** Matteo Carpi

## Brief history

The Pittsburgh Sleep Quality Index (PSQI) was first developed in 1989 by David J. Buysse and colleagues to assess sleep quality, particularly in clinical populations, with a focus on individuals with psychiatric disorders [1]. At the time, the authors noted that, despite the high frequency of sleep complaints associated with such conditions, there was a lack of standardized instruments to measure sleep quality and disturbances. Since its introduction, the PSQI has become widely adopted in both research and clinical settings, within and outside the fields of psychiatry and sleep medicine [2].

Being largely employed in diverse clinical samples, as well as in the general population and epidemiological studies, the questionnaire is now unanimously recognized as the most common subjective measure of sleep quality. As of October 2024, the original 1989 study has been cited 37 666 times on Google Scholar and 23 78 times on Scopus, and the PSQI is frequently mentioned as a key tool for clinical assessment in leading sleep medicine textbooks [3,4].

## Description

The PSQI is a 19-item self-report questionnaire comprising both fixed-choice and open-ended questions, evaluating several aspects of sleep over the past month, including perceived sleep quality, sleep habits (e.g. habitual bedtimes and wake times) and sleep disturbances (e.g. difficulty falling asleep, disordered breathing, nightmares and pain). Five additional questions, rated by a habitual bed partner, assess aspects of sleep that are difficult to self-report (e.g. loud snoring, pauses in breathing, legs twitching and disorientation during sleep) and may be administered for clinical assessment but are not included in the scoring.

A dedicated scoring procedure, described in the original validation study [1], yields scores for seven distinct components: (1) subjective sleep quality, (2) sleep latency, (3) sleep duration, (4) habitual sleep efficiency, (5) sleep disturbances, (6) use of sleeping medication and (7) daytime dysfunction. Component scores range from 0 (no difficulty) to 3 (severe difficulty), and their sum produces a global score ranging from 0 to 21, with higher values indicating poorer sleep quality. A PSQI score greater than 5 is considered indicative of poor sleep quality, based on its high sensitivity and specificity in identifying patients with sleep complaints.

The appendix of Buysse *et al*.’s study [1] includes the PSQI item format and scoring sheet, which, along with detailed guidelines on the instrument’s use, are also accessible via the University of Pittsburgh’s Center for Sleep and Circadian Science website [5].

To date, the questionnaire has been translated into more than 60 languages, facilitating its global dissemination.

## Validity

The psychometric and clinimetric properties of the PSQI have been extensively studied, with numerous studies applying the instrument in a wide range of clinical conditions, as well as in non-clinical samples.

In their validation study involving 148 participants (*n* = 52 healthy controls with no sleep complaints, *n* = 34 patients with major depressive disorder, and *n* = 62 patients with sleep disorders), Buysse *et al.* [1] found good internal consistency for the PSQI global score, with a Cronbach’s alpha coefficient of 0.83, and high stability over time (i.e. test–retest reliability), with *r* = 0.85. They also confirmed the instrument’s criterion validity by demonstrating its ability to differentiate subjects with disturbed sleep (i.e. patients experiencing difficulty initiating or maintaining sleep, excessive somnolence, or depression) from good sleepers. Differences were observed between patients and controls in both the global score and component scores, and the cut-off score of 5 correctly identified up to 89% of patients, with a sensitivity of 90% and specificity of 87%.

Subsequent studies have confirmed these findings in diverse clinical and non-clinical groups [6,7], also supporting the questionnaire’s convergent validity, as shown by significant correlations with sleep variables derived from daily sleep diaries and subjective ratings of depressive symptoms [7].

More recently, a comprehensive systematic review and meta-analysis summarized the evidence on the PSQI’s psychometric properties, with a particular focus on its dimensionality and factor structure [2]. While further rigorous studies may be needed, a one-factor model generally fits poorly, and two- or three-factor models are now considered more appropriate based on the current research. In some studies, the sleeping medication and daytime dysfunction components have shown poor factor loadings. However, given the good reliability of the global score across settings, it remains a practical measure of overall sleep quality for large samples, whereas single items and components may be better suited to clinical assessment and decision-making, given their content validity and coverage of key sleep quality domains [2].

## Key research

In addition to its wide application in clinical research [2], the PSQI has been employed in numerous studies investigating sleep quality and its determinants across general population samples in diverse cultural contexts. For example, in a community sample of 9248 German adults, the prevalence of poor sleep (PSQI > 5) was 36%, with females reporting more sleep problems than males [8]. In this study, poor sleep was significantly associated with higher levels of fatigue, physical complaints, anxiety and reduced quality of life.

Similarly, a prevalence of 41% was found in a nationwide sample of 165 193 Korean adults, with poor sleep quality being linked to lower socioeconomic status, poor health habits and worse mental health [9].

Of particular relevance to occupational health research, the PSQI has been widely used to assess sleep quality across various working populations, including healthcare professionals [10], shift workers [11] and military personnel [12]. These studies consistently demonstrate the impact of poor sleep on fatigue, cognitive performance and mental health, highlighting the PSQI’s utility in understanding sleep-related challenges in the workplace.
