# Correction to: The Pittsburgh Sleep Quality Index: a brief review

**DOI:** 10.1093/occmed/kqaf038

**Published:** 2025-05-09

This is a correction to: Matteo Carpi, The Pittsburgh Sleep Quality Index: a brief review, *Occupational Medicine*, Volume 75, Issue 1, January 2025, Pages 14–15, https://doi.org/10.1093/occmed/kqae121

The following changes have been made to the originally published manuscript. The author’s name has been emended in the article to read: “Matteo Carpi” instead of: “Gail Kinman”.

